# Enhancer Rewiring Orchestrates Inflammation and Loss of Cell Identity During Muscle Stem Cell Aging

**DOI:** 10.1111/acel.70289

**Published:** 2025-11-14

**Authors:** Changyou Shi, Na Yang, Evelyn Pizano, Lin Wang, James R. Occean, Chang‐Yi Cui, Ling Liu, Christopher Dunn, Frimpong Boadu, Qiong Meng, Nirad Banskota, Jen‐Hao Yang, Jinshui Fan, Supriyo De, Jianlin Cheng, Thomas A. Rando, Vittorio Sartorelli, Payel Sen

**Affiliations:** ^1^ Laboratory of Genetics and Genomics, National Institute on Aging NIH Baltimore Maryland USA; ^2^ Department of Neurology and Broad Stem Cell Research Center University of California, Los Angeles Los Angeles California USA; ^3^ Flow Cytometry Unit, National Institute on Aging NIH Baltimore Maryland USA; ^4^ Department of Electrical Engineering and Computer Science University of Missouri Columbia Missouri USA; ^5^ Computational Biology and Genomics Core, Laboratory of Genetics and Genomics, National Institute on Aging NIH Baltimore Maryland USA; ^6^ Laboratory of Muscle Stem Cells and Gene Regulation, National Institute of Arthritis and Musculoskeletal and Skin Diseases NIH Bethesda Maryland USA

**Keywords:** adult stem cells, aging, chromatin, epigenome, multi‐omics, muscle

## Abstract

Loss of regeneration is a key feature of aging organs, often linked to stem cell exhaustion. Skeletal muscle stem cells (MuSCs) undergo age‐related numerical and functional decline, contributing to reduced regenerative potential. Using low‐input multi‐omics, we systematically profiled the epigenome, transcriptome, and 3D genome of MuSCs from individual mice across 3 age groups (young, old, and geriatric) and both sexes. At baseline, young male MuSCs showed reduced expression of cell cycle‐related mRNAs. In aged mice, particularly males, MuSCs exhibited early alterations (emerging during the transition from young to old age) including enhanced proinflammatory signaling, and loss of cell identity. Late alterations (emerging during the transition from old to geriatric age) included heightened inflammation, widespread enhancer activation, and extensive 3D genome rewiring. Proinflammatory pathways were enriched for interferon signaling and correlated with endogenous retroviral expression and NFκB activity. Late‐stage epigenome and 3D genome rewiring reflected downstream degenerative changes in muscle organization, response to cytokines, and loss of myogenic identity. Thus, progressive molecular shifts may explain the aggravated proliferative deficit and functional impairment observed in MuSCs during aging.

## Graphical abstract

A comprehensive multi‐omic profiling of murine muscle stem cells across three ages and both sexes reveals extensive enhancer and 3D genome rewiring, concomitant with increased inflammation, lineage drift, loss of quiescence, and reduced myogenic potential.

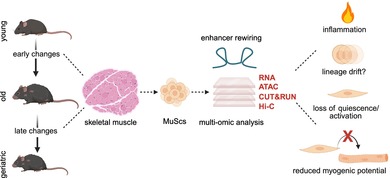

## 1 Introduction

Skeletal muscles (SKM) comprise 40%–50% of body mass and represent a class of tissues under voluntary control of the somatic nervous system. With age, there is a reduction in regenerative capacity and a progressive loss of SKM mass (sarcopenia) which leads to functional decline, frailty, and increased mortality (Sayer et al. 2024). Age‐related decline in regenerative potential in the SKM is attributed to the exhaustion of adult stem cells known as muscle stem cells (MuSCs) or satellite cells which reside between the sarcolemma and basal lamina (Munoz‐Canoves et al. 2020; Yin et al. 2013). Ablation studies have established MuSCs as chief myogenic progenitors (Lepper et al. 2011; McCarthy et al. 2011; Murphy et al. 2011; Sambasivan et al. 2011). In the absence of injury, MuSCs remain in a state of quiescence characterized by low metabolic activity and expression of PAX7, and other stemness markers such as NOTCH1‐3, CD34 and CXCR4 (Sousa‐Victor et al. 2015; Yin et al. 2013). Injury triggers a coordinated series of events starting with MuSC activation, and cell division. Some of the activated MuSCs (ASCs) produce myoblasts which differentiate into myocytes and fuse to form myotubes while the remaining ASCs return to quiescence to maintain the stem cell pool. Myotubes further mature into myofibers, with the migration of nuclei from a central to a peripheral position (Kuang et al. 2007).

With age, there is both a numerical and functional decline in MuSCs, the reasons for which are only partially understood (Brack and Munoz‐Canoves 2016). Age‐related numerical exhaustion may be attributed to escape from quiescence in part triggered by release of fibroblast growth factor 2 (FGF2) from aged myofibers (Chakkalakal et al. 2012), activation of mammalian target of rapamycin complex 1 (mTORC1) and AKT signaling (Rodgers et al. 2014), decline in anti‐aging KLOTHO expression (Ahrens et al. 2018; Sahu et al. 2018), mitotic catastrophe (Liu et al. 2018), or senescence (Moiseeva et al. 2023; Sousa‐Victor et al. 2014). By contrast, age‐related functional decline occurs due to specific cell‐extrinsic (e.g., microenvironment, mechanical stress, senescent niche) and cell‐intrinsic (e.g., DNA damage accumulation, metabolic changes, epigenetic alterations) mechanisms (Brack and Rando 2007). Here, we focus on decoding the intricate cell‐intrinsic epigenomic reprogramming events that underpin functional deficits in aged MuSCs.

Technical limitations related to low cell numbers compounded by the numerical decline of MuSCs with age, have posed serious challenges in integrating multiple modalities while also estimating biological variability and sex differences. Due to its sensitivity, the assay for transposase‐accessible chromatin using sequencing (ATAC‐seq), has been the easiest to implement (Grandi et al. 2022), and has revealed accessibility changes with age (Dong et al. 2022; Lazure et al. 2023; Shcherbina et al. 2020). Lazure et al. (2023) further reported an inverse relationship between chromatin accessibility and DNA modification as measured by whole genome bisulfite sequencing. Inferred transcription factor (TF) activity from RNA‐seq and ATAC‐seq has pinpointed several key players in stem cell identity and myogenesis (Chow et al. 2021; Garcia‐Prat et al. 2020). Liu et al. (2013) profiled histone modifications (H3K27me3, H3K4me3, and H3K36me3) by ChIP‐seq in young and old MuSCs pooled from multiple animals. The most remarkable change was in H3K27me3, which accumulated and spread in old MuSCs, particularly over histone genes with reduced expression (Hu, Chen, et al. 2014; O'Sullivan et al. 2010). A more recent report showed reduced levels of constitutive heterochromatin markers H3K9me2/3 and HP1 in old MuSCs via microscopy (Kang et al. 2024). Finally, two studies reported extensive rewiring of local contacts in aged MuSCs using high‐throughput chromosome conformation capture (Hi‐C) (Yang et al. 2023; Zhao et al. 2023). However, prior studies have rarely examined dynamic chromatin marks like H3K27ac in the context of MuSC aging, and few have addressed sex differences or incorporated a broader multi‐omic framework.

To address the abovementioned gaps, we have employed sensitive low‐input multi‐omic methods to carefully profile the epigenomic, transcriptomic, and 3D genomic landscape in freshly isolated MuSCs (FISCs) from individual mice across three age groups and both sexes. Our integrated findings from four modalities reveal distinct age‐related temporal events, with the transcriptome and accessible chromatin showing early signs of pro‐inflammatory alterations. In later stages, we find evidence of profound rewiring of the enhancer landscape, and sustained inflammation which may drive downstream degenerative changes.

## 2 Results

### 2.1 FISCs Show Sex‐Biased Quantitative and Qualitative Decline With Age

**FIGURE 1:**
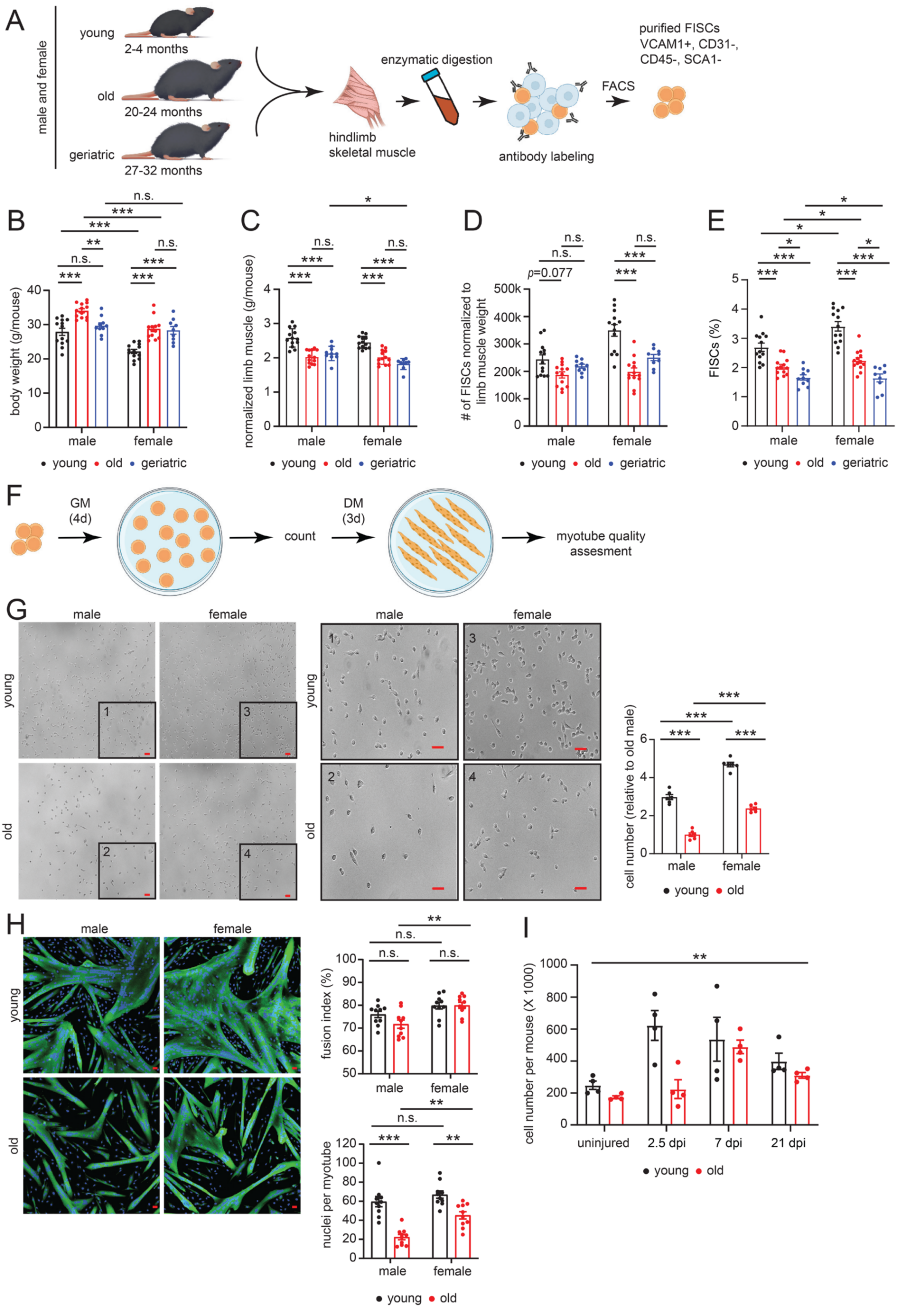
FISCs show a sex‐biased quantitative and qualitative decline with age. (A) Schematic of the experiment in which hindlimb muscles from young (2–4 months), old (20–24 months), and geriatric (27–32 months) mice were collected. Mononucleated cells were dissociated from limb muscles using physical and enzymatic methods, and FISCs (identified as VCAM+ CD31− CD45− SCA1−) were isolated using FACS. (B) Comparison of body weight, (C) body weight normalized hind limb muscle mass, (D) muscle mass normalized FISC numbers, and (E) the percentage of FISCs in total mononucleated cells isolated from young, old, and geriatric mice (data represented as mean ± SEM, *n* = 9–13 mice). Significance was calculated using two‐way ANOVA with Tukey's multiple comparisons test: *p < 0.05, **p < 0.01, ***p < 0.001. (F) Schematic of MuSC proliferation and differentiation in vitro. (G) Sorted FISCs were seeded in myogenic growth medium (GM) and cultured for 4 days, after which cells were counted under brightfield microscopy using ImageJ. Scale bar represents 20 μm. Magnified insets are in the middle panel. Bar plot on the right shows the quantification of proliferating cells from young and old mice of both sexes (data represented as mean ± SEM, *n* = 6 from 2 mice with 3 wells per mouse) represented relative to old male. Significance was calculated using two‐way ANOVA with Tukey's multiple comparisons test: ***p < 0.001. (H) Sorted FISCs were seeded in GM and cultured for 4 days before switching to myogenic differentiation medium (DM). After 3 days in DM, cells were fixed and immunostained for myosin heavy chain (MHC, green), with nuclei stained using DAPI (blue). Scale bar represents 20 μm. The bar plot on the right shows quantification of myotube formation, including the fusion index (percentage of nuclei within myotubes out of the total nuclei) and the number of nuclei per myotube, data represented as mean ± SEM, *n* = 10 from 2 mice with 5 images per mouse. Significance was calculated using two‐way ANOVA with Sidak's (for fusion index), and Tukey's (for nuclei per myotube) multiple comparisons test: **p < 0.01, ***p < 0.001. (I) Total number of FISCs recovered per mouse at indicated days after injury, data represented as mean ± SEM, *n* = 4 mice. Significance was calculated using two‐way ANOVA with Sidak's multiple comparisons test: **p < 0.01. For panels (B–H), both males and females were considered, for (I) only male was used.

To investigate MuSC function during aging we sourced male and female C57BL/6JN mice spanning three age groups: young (2–4 months), old (20–24 months), and geriatric (27–32 months) from the NIA rodent colony (see Section 4). MuSCs were freshly isolated (FISCs) from hind limb SKM, either from injured or uninjured tissue using fluorescence activated cell sorting (FACS) following established protocols and previously defined markers, VCAM1+, CD31−, CD45−, and SCA1− (Liu et al. 2015) (see Section 4, Figure 1A and Figure S1A–E). Greater than 95% of the sorted cells were also positive for the known MuSC marker, PAX7 (Figure S1F). While body weight increased in old mice, there was a modest decline in geriatric males (Figure 1B). Hind limb muscle mass (normalized to body weight) showed a significant decrease with age (Figure 1C). The number of FISCs recovered per mouse (normalized to hind limb muscle mass) decreased significantly in female but not male mice (Figure 1D). FISCs estimated as % of total mononuclear cells from uninjured SKM showed a significant decrease with age in both sexes (Figure 1E), in line with previous reports (reviewed in Brack and Munoz‐Canoves 2016).

To test for function, we seeded equal numbers of FISCs from young and old mice in a growth medium (GM) containing human fibroblast growth factor (hFGF) for 4 days and then changed to a differentiation medium (DM) for 2 days (see Section 4, Figure 1F). Old FISCs from both sexes showed reduced proliferation (Figure 1G) and myotube formation (Figure 1H), evident from myosin heavy chain (MHC) expression, a marker for mature myotubes. Myotubes from old FISCs were visibly thinner, shorter, and fewer (Figure 1H). Notably, the fusion index (number of nuclei inside MHC+ myotubes divided by the total number of nuclei) was unaltered with age. In contrast, the nuclei per myotube were significantly reduced in the old (Figure 1H bar plots). Collectively, these observations indicate that a proliferation rather than fusion defect dictates poor myotube formation with age. Interestingly, we found that male FISCs showed less proliferation than females regardless of age (Figure 1G).

We injected 1.2% BaCl_2_ into the hind limb SKM of young and old mice to induce injury and measured MuSC numbers from injured muscles over a 21‐day period using FACS. In line with an in vitro proliferative defect, we recovered fewer FISCs 2.5 days post‐injury from the muscles of old mice, when young FISCs showed peak proliferation (Figure 1I).

Overall, we confirmed the well‐established quantitative and qualitative decline of MuSCs with age (Sousa‐Victor et al. 2015), but further demonstrated that this decline is predominantly driven by a proliferative defect, with a pronounced impact in males.

### 2.2 Bulk Transcriptomic Profiles Highlight Aggravated Aging in Geriatric FISCs

**FIGURE 2:**
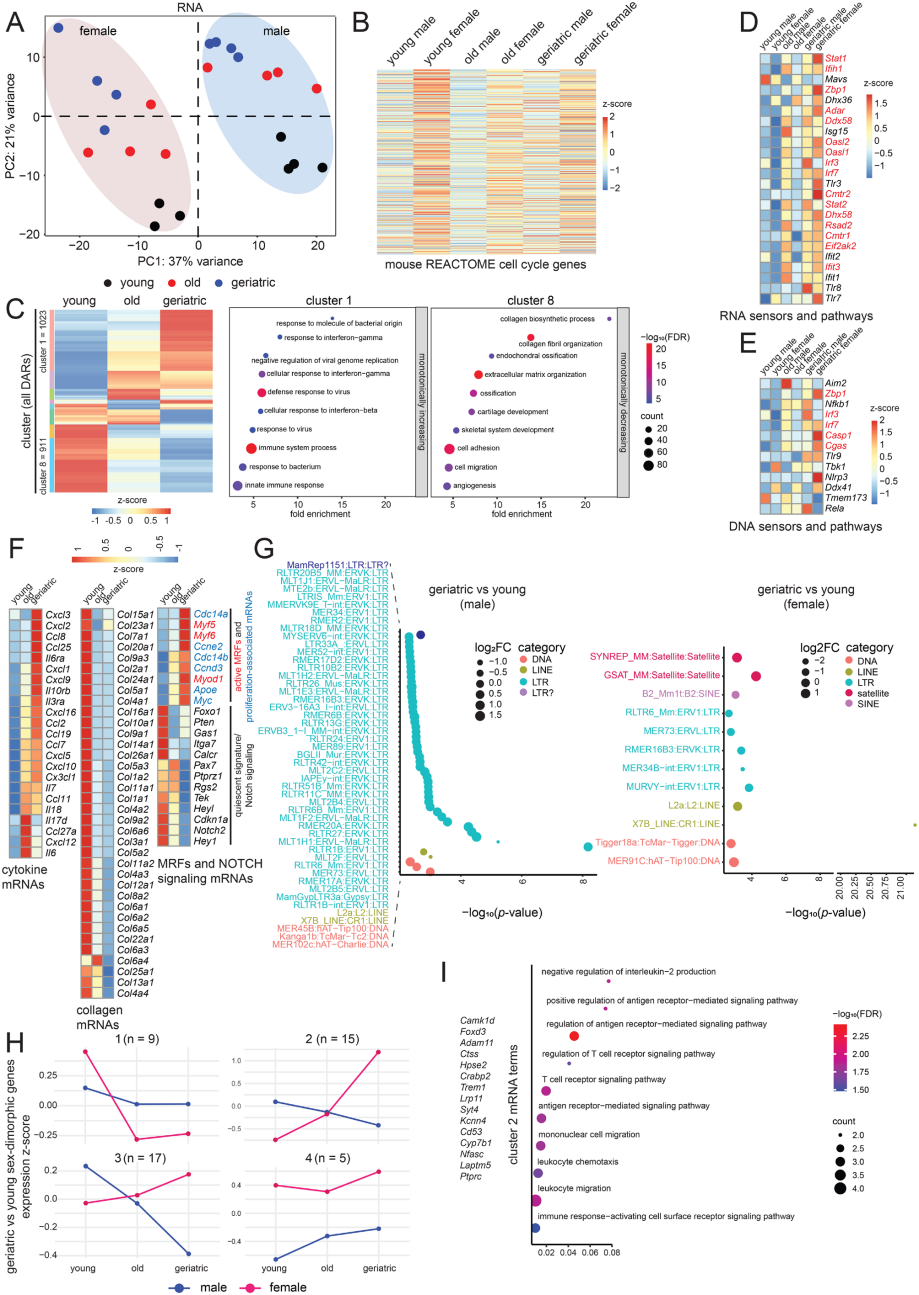
Bulk transcriptomic profiles highlight aggravated aging in geriatric FISCs. (A) PCA plot of RNA‐seq data from young, old, and geriatric FISCs, *n* = 7–8 mice per age group. (B) Heatmap of mouse REACTOME cell cycle‐related mRNAs across age and sex. (C) Clustered heatmap of count mean values in young, old, and geriatric FISCs for all DARs (FDR < 0.05). Clusters are identified by color on the left. Bubble plots of biological process GO terms for age‐related mRNAs showing a monotonic increase (cluster 1) and monotonic decrease (cluster 8). Bubble size represents the number of mRNAs in each term, while bubble color indicates the enrichment significance. (D) Heatmap of select RNA sensors and RNA sensing pathway mRNAs. (E) Heatmap of select DNA sensors and DNA sensing pathway mRNAs. (F) Expression heatmaps of cytokine mRNAs (left), collagen mRNAs (middle), myogenic regulatory factors (MRFs, right, red), proliferation‐associated mRNAs (right, blue), and quiescent signature/Notch signaling mRNAs (right, black). For (C–F), the row *z*‐score color scale is shown. (G) Bubble plot of TE category significantly changed in geriatric compared to young in male (left) and female (right). Color indicates the category, and size indicates the log_2_FC of the specific TEs. (H) Line plots showing average *z*‐scored expression patterns for sex‐dimorphic mRNAs across age. (I) Bubble plots of the top enriched biological process GO terms for cluster 2 in (H). The mRNAs in cluster 2 are indicated on the left. For panels (A–I), both males and females were considered.

To assess transcriptomic changes in FISCs during aging, we adopted the SMART‐seq (Switching Mechanism at 5′ End of RNA Template) methodology to generate full length cDNA libraries for bulk low‐input RNA‐seq from young (*n* = 7), old (*n* = 8), and geriatric (*n* = 8) mice (see Section 4, Table S1). Replicate reproducibility is shown in Figure S2A. Principal Component Analysis (PCA) revealed that the primary sources of variation in the transcriptomic data were sex (PC1) and age (PC2) (Figure 2A). The variation between sexes was driven by mRNAs transcribed from genes located on the sex chromosomes, such as *Ddx3x*, *Kdm6a*, *Kdm5d*, *Uty*, *Ddx3y*, etc. (Figure S2B); however, this set was not the focus of the present study. Of greater interest, and in concordance with the proliferation defect in male MuSCs (Figure 1G), we observed relatively lower expression of mRNAs encoding cell cycle‐related proteins in males (Figure 2B). Notably, this difference was evident even in young mice. We next used DESeq2 (Love et al. 2014) to identify differentially abundant mRNAs (DARs) across age groups. A 2‐way comparison across ages revealed approximately equal numbers of mRNAs that increased or decreased with age (Figure S2C, Table S2). When visualizing all DARs in a 3‐way comparison, the majority either increased (Figure 2C cluster 1, Table S2) or decreased (Figure 2C cluster 8, Table S2) monotonically (*n* ~ 1000 in each category), with the geriatric group showing the most pronounced changes.

A Gene Ontology (GO) analysis (Huang da et al. 2009) of the DARs revealed enrichment in inflammation‐related pathways in cluster 1 and collagen and extracellular matrix (ECM) related pathways in cluster 8 (Figure 2C bubble plots). These same pathways were captured independently in a Gene Set Enrichment Analysis (GSEA; Mootha et al. 2003, Figure S2D–F). Given the strong prevalence of mRNAs encoding proteins involved in the antiviral response and type I and II interferon pathways, we interrogated known mRNAs in these pathways in greater detail. We found increased expression with age of predominantly RNA sensors (Figure 2D) and, to a lesser extent, DNA sensors (Figure 2E), along with corresponding downstream signaling. These sensors are a class of pattern recognition receptors (PRRs) that bind nucleic acids (normally from foreign pathogens) as ligands and trigger innate immune signaling (Li and Wu 2021). Hyperactivation of PRRs is increasingly being recognized as a feature of inflammaging (Walker et al. 2022). The mRNAs colored in red in Figure 2D,E were also found in cluster 1 (Figure 2C). Consistent with the proliferation defect in male MuSCs (Figure 1G), we found early expression of the RNA and DNA sensors in the old male (but not old female) group (Figure 2D,E).

We present heatmaps of normalized RNA mean counts (Figure 2F), example genome browser snapshots (Figure S3A,B), and normalized RNA counts from individual animals (Figure S3C,D) for several other mRNA groups. Notably, 36 of the 44 collagen mRNAs in the mouse genome decreased with age (Figure 2F). Additionally, we noted that mRNAs encoding active myogenic regulatory factors (MRFs) and some proliferation‐associated factors were increased, while those related to Notch signaling and quiescent signatures were decreased suggesting a general activated state for aged FISCs that is in line with previous reports (Figure 2F) (Dong et al. 2022). We validated these transcriptomic findings at the protein level using a multiplexed LASER bead assay from conditioned media of MuSC cultures (Figure S2G), and by quantitative immunofluorescence (IF) of collagens (Figure S2H).

We further interrogated the expression of transposable elements (TEs), which are known to increase in abundance with age and trigger an inflammatory response via the cGAS‐STING, interferon, and NFκB pathways (Chen et al. 2016; Di Giorgio and Xodo 2022). Notably, the interferon pathway was strongly enriched in aged FISCs in GSEA (Figure S2D–F). We implemented multi‐mapping and an expectation–maximization algorithm via TEtranscripts (Jin et al. 2015) which probabilistically distributes reads across repeat elements. Our results showed that 47 TEs in geriatric male FISCs and only 9 TEs in geriatric female FISCs were elevated compared to young (Figure 2G). The TE categories in males were primarily of the endogenous retrovirus (ERV) class, which, although cannot autonomously transpose, can still provoke an inflammatory response via RNA and DNA sensing mechanisms (Chen et al. 2016; Di Giorgio and Xodo 2022).

To detect mRNAs with sexually dimorphic changes with age, we fit a DESeq2 model with an age‐by‐sex design (~age*sex). We obtained the greatest number of age‐related sex‐dimorphic mRNAs in the geriatric vs. young comparison (Figure S2I). We clustered the 46 resulting differentially expressed mRNAs into four groups to identify dominant patterns (Figure 2H, Table S2) and then performed pathway enrichment analysis. Interestingly, cluster 2, which constitutes a subset of mRNAs that increases with age in females but shows the opposite trend in males (*n* = 15), was enriched for immune signaling and leukocyte trafficking pathways (Figure 2I). These results point to divergent, sex‐dependent immune and inflammatory programs during MuSC aging.

Taken together, aged FISCs exhibit a strong pro‐inflammatory mRNA signature, particularly in the geriatric group, which may be partially triggered by cell‐intrinsic ERV expression. By contrast, collagen and ECM mRNAs were strongly decreased with age.

### 2.3 Chromatin Accessibility Increases at Enhancer Regions in Aged FISCs

**FIGURE 3:**
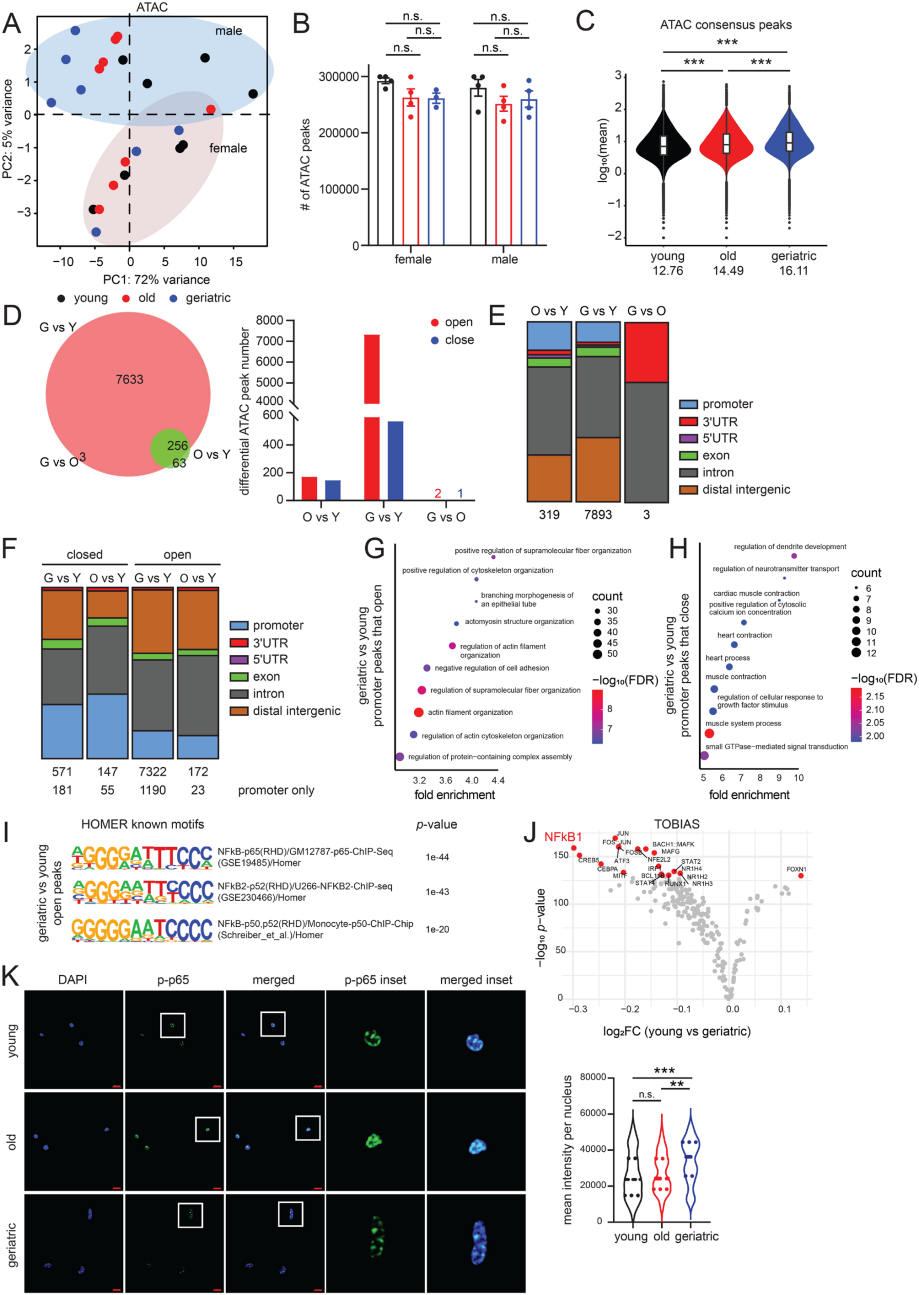
Chromatin accessibility increases at enhancer regions in aged FISCs. (A) PCA plot of ATAC‐seq data from young, old, and geriatric FISCs. (B) The total number of ATAC peaks called in young, old, and geriatric FISCs, data represented as mean ± SEM, *n* = 7–8 mice per age group. Significance was calculated using two‐way ANOVA with Sidak's multiple comparisons test and revealed no statistically significant differences. (C) Violin plot showing the distribution of mean intensity of consensus ATAC peaks in young, old, and geriatric FISCs. Median values are indicated below. ***p < 0.001 by an unpaired two‐tailed Welch's *t*‐test. (D) (Left) Venn diagram showing the overlap of differential ATAC peaks across 3 comparisons: Old vs. young (O vs. Y), geriatric vs. old (G vs. O), and geriatric vs. young (G vs. Y). (Right) Bar plot showing differential ATAC peaks split by “open” or “close” status across the same 3 comparisons. (E) ChIPseeker annotation of the differential ATAC peaks identified in (D) across the 3 comparisons. Peak numbers are indicated at the bottom. (F) ChIPseeker annotation of differential ATAC peaks split by “open” or “close” status in two comparisons (old vs. young or geriatric vs. young). Peak numbers are indicated at the bottom. (G) GO analysis of the nearest genes identified in the promoter category in (F) in the geriatric vs. young “open” group. (H) Same as (G) except for geriatric vs. young “close” group. (I) HOMER analysis showing top 3 known TF motifs associated with peaks that open in the geriatric vs. young group. (J) Volcano plot of differential TF footprinting analysis using TOBIAS in the geriatric vs. young group, NFκB is highlighted in a larger font. (K) IF images of young, old, and geriatric FISCs stained with phosphorylated NFκB p65 (p‐p65) antibody. Images are at 63× magnification. Scale bar represents 10 μm. Enlarged insets and mean signal intensity per cell is shown on the right. Significance was calculated using one‐way ANOVA with Tukey's multiple comparisons test: **p < 0.01, ***p < 0.001 from ~10 images (at 20× magnification) per mouse. For panels (A–I), both males and females were considered, for (K) only male was used.

To establish a chromatin basis for transcriptomic changes during MuSC aging, we performed ATAC‐seq (Grandi et al. 2022) in FISCs from young (*n* = 8), old (*n* = 8), and geriatric (*n* = 7) mice (see Section 4, Table S1). Replicate reproducibility is shown in Figure S3E. Similar to our transcriptomics data, PCA of ATAC‐seq profiles identified age and sex as the primary sources of variation (Figure 3A), although their relative contributions were reversed. PC1 accounted for 72% of the variance and clearly separated males by age, whereas females showed weaker separation along this axis. Given that age explained substantially more variance than sex, we prioritized age effects in subsequent analyses.

There were no statistical differences in the total number of ATAC peaks identified in each age group (Figure 3B), although there was a progressive increase in accessibility with age (Figure 3C). A differential peak analysis using DiffBind (Stark and Brown 2011) identified 7893 peaks that were significantly different in geriatric compared to young (Figure 3D left, Table S3), with a majority of them (7322) gaining accessibility (opening) with age and only 571 losing accessibility (closing, Figure 3D right, Table S3). Most of these differential peaks were annotated to intronic and distal intergenic regions suggesting they could function as enhancers (Figure 3E). A minor subset of promoter peaks that were proximal to genes were evident when annotating the peaks that open or close with age separately (Figure 3F). We performed a pathway enrichment analysis of these promoter‐proximal genes in the geriatric vs. young comparison. Genes near peaks that opened with age were enriched for actin cytoskeleton/filament organization pathways (Figure 3G); while genes near peaks that closed with age were enriched for muscle system and contractile functions (Figure 3H), consistent with reduced myogenic potential. Despite these trends, overall, there was only a weak positive correlation between ATAC and RNA signal (Figure S3F), likely because differential ATAC peaks were primarily in intronic/distal intergenic rather than promoter regions.

We then focused our efforts on the discovery of TF motifs from the chromatin accessibility patterns. First, we performed a simple static motif enrichment analysis using HOMER (Heinz et al. 2010) on the differential ATAC peaks that open in the geriatric vs. young comparison. The top 3 TF motifs were all NFκB sites (Figure 3I). Second, we implemented TOBIAS (Bentsen et al. 2020) footprinting analysis which integrates local chromatin accessibility changes and incorporates them into TF activity predictions. TOBIAS's robust statistical approach also enables cross‐condition comparisons and identified NFκB1 (p50) as having significantly different TF activity during aging (geriatric vs. young comparison shown in Figure 3J). Since NFκB1 lacks a transactivation domain, it usually heterodimerizes with p65 (RelA) to stimulate transcription (Zhang et al. 2017). To validate TF activity findings from TOBIAS, we performed IF of phospho‐p65 (Ser 536), a marker for active NFκB signaling. We found significantly higher signal in geriatric FISCs with evidence of nuclear localization (Figure 3K, note the more central punctate signal in insets).

In sum, the ATAC‐seq profiles in aging MuSCs suggest that the chromatin landscape is substantially reshaped at enhancers that could potentiate a pro‐inflammatory transcriptome via NFκB activity. By contrast, ATAC accessibility changes at promoters imply substantial actin filament reorganization and downregulation of muscle‐related functions.

### 2.4 Combinatorial Analyses of Histone Modifications and 3D Contacts Show Enrichment of Enhancers States

**FIGURE 4:**
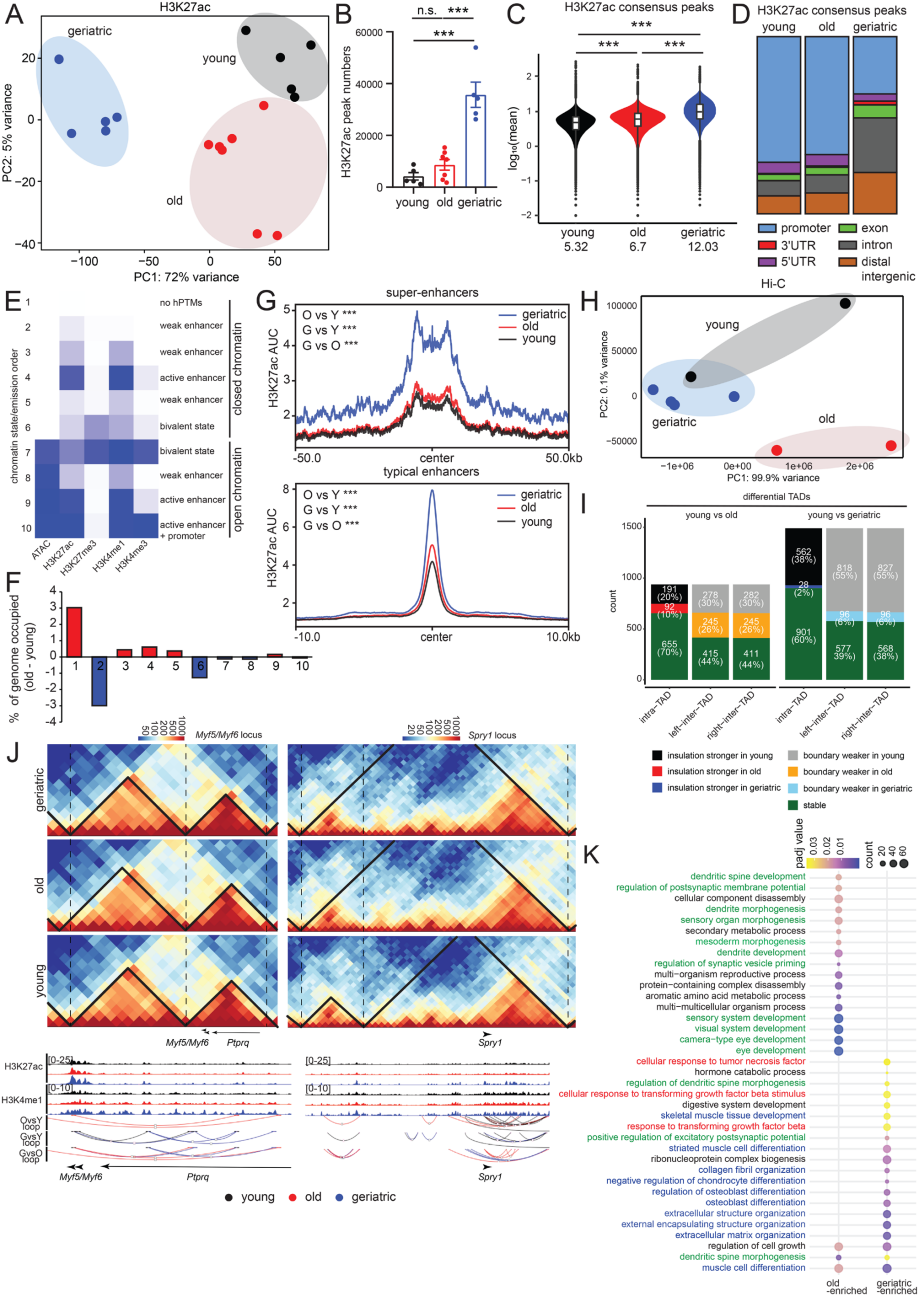
Combinatorial analyses of histone modifications and 3D contacts show enrichment of enhancer states. (A) PCA plot of H3K27ac data from young, old, and geriatric FISCs. (B) Bar plot showing the number of H3K27ac peaks in young, old, and geriatric FISCs, data represented as mean ± SEM, *n* = 5–7 mice. Significance was calculated using one‐way ANOVA with Tukey's multiple comparisons test: ***p < 0.001. (C) Violin plot showing the distribution of mean intensity of consensus H3K27ac peaks in young, old, and geriatric FISCs. Median values are indicated below. ***p < 0.001 by an unpaired two‐tailed Welch's *t*‐test. (D) ChIPseeker annotation of H3K27ac consensus peaks in young, old, geriatric FISCs. (E) ChromHMM modeling of chromatin states (row) in young and old FISCs. The heatmap of emission parameters represents enrichment probabilities, with deeper color indicating a higher probability. (F) Bar plot showing genomic occupancy of each state. A positive value represents increased genome occupancy in old while a negative value represents increased genome occupancy in young. (G) Metaplot of H3K27ac signal over super enhancers (top) and typical enhancers (bottom) in young, old, and geriatric FISCs. ***p < 0.001 by an unpaired two‐tailed Welch's *t*‐test. (H) PCA plot of KR normalized Hi‐C contact matrices at 1 mb resolution. (I) Stacked bar plot showing total and differential TAD changes for indicated age comparisons. (J) Visualization of differential TADs and loops at the *Myf5*/*Myf6* locus (left) and the *Spry1* locus (right). TADs are indicated with solid black lines while boundaries are indicated with a dashed black line. Color scale indicates log transformed contact frequency. For panels (A–G), both males and females were considered, for (H–K) Hi‐C data were from males only. (K) Bubble plot visualization of functional pathway enrichments (biological process) from ABC outputs. The top 20 pathways in old vs. young and geriatric vs. young are reported with bubble size representing gene counts and color scale the FDR *q*‐value.

Recognizing the limitations of RNA‐seq and ATAC‐seq in fully capturing epigenetic complexity, we pursued an integrative approach to incorporate additional epigenetic modalities. Since ATAC‐seq provided clues about enhancer restructuring (Figure 3E), we performed Cleavage Under Targets and Release Using Nuclease (CUT&RUN) (Skene and Henikoff 2017) for two enhancer marks, H3K4me1 and H3K27ac, in FISCs from young (*n* = 5–6), old (*n* = 7–9), and geriatric (*n* = 4–5) mice (see Section 4, Table S1). Replicate reproducibility is shown in Figure S4A,B. Similar to the ATAC data, the primary source of variation (PC1) was age (Figure 4A, Figure S4C), suggesting that chromatin‐level information tracks with the age of stem cells, irrespective of modality. By contrast, transcriptomic differences were primarily associated with sex, followed closely by age (Figure 2A). Interestingly, for both H3K27ac and H3K4me1, the young and old data clustered together, while the geriatric samples were more distinct, indicating a more robust enhancer restructuring in the later stages of life. We also identified significantly higher H3K27ac peaks in geriatric FISCs (Figure 4B), while H3K4me1 showed an upward but statistically non‐significant trend (Figure S4D). The median peak signal for both modifications increased with age (Figure 4C, Figure S4E). As expected, H3K4me1 peaks were mostly annotated to distal intergenic and intronic regions (Figure S4F). Interestingly, H3K27ac peaks annotated mainly to promoters in the young and old but to distal intergenic and intronic regions in geriatric FISCs (Figure 4D).

We next combined our ATAC and CUT&RUN data for enhancer marks with published ChIP‐seq data for H3K4me3 and H3K27me3 (Liu et al. 2013) and defined 10 chromatin states using ChromHMM (Ernst and Kellis 2012). In the context of ChromHMM, “chromatin state” is a probabilistic inference of a functional annotation (promoter, enhancer, etc.) for each genomic bin. Notably, this analysis was performed in young and old FISCs, as the published dataset did not include a geriatric group. ChromHMM is superior to our peak calling approach (in Figure 4B–D), because it “learns” de novo chromatin states from recurring combinations of modifications and accessibility genome‐wide. Using ChromHMM, we uncovered multiple enhancer‐enriched states (Figure 4E). States 3–5 and 9, marked by classic enhancer markers, H3K4me1 and H3K27ac, showed increased genome occupancy in old FISCs (Figure 4F). We further classified the enhancers by rank ordering of super‐enhancers (ROSE) algorithm (Whyte et al. 2013) to identify super‐enhancers and typical enhancers. Using either H3K27ac (Figure 4G), or H3K4me1 (Figure S4G), aged FISCs, especially geriatric, showed greater signal intensity at both enhancer classes (Table S4). Overall, these approaches revealed greater activity at enhancer elements during MuSC aging.

We surmised that the increased enhancer activity with age is indicative of a reorganized 3D genome. Indeed, a previous report noted several age‐related 3D genome rewiring events including altered compartmentalization, loss of insulation at topologically‐associated domains (TADs), and age‐specific loops (Zhao et al. 2023). We performed Hi‐C in young (*n* = 2), old (*n* = 2), and geriatric (*n* = 4) male mice (with MuSCs pooled from 3 to 4 animals per replicate, Table S1). PCA of Hi‐C data revealed that samples clustered distinctly by age, with nearly all variance explained by PC1 (Figure 4H). Replicate reproducibility is shown in Figure S4H. Our Hi‐C libraries were sequenced to a depth of ~1.14B reads per sample, with 88%–90% of non‐duplicated reads representing valid high‐quality, Hi‐C contacts (Table S4), thus enabling differential TAD and loop analysis with robust statistical tests. We identified TADs at an FDR threshold of 0.001 in each age group, yielding 2261 TADs in the young group, 2279 in the old group, and 1877 in the geriatric group. For differential analysis, we used the young TADs (*n* = 2261) as reference and evaluated differences across intra‐TAD, left inter‐TAD, and right inter‐TAD regions. A TAD was considered differential if at least one of these regions showed a statistically significant change (*p* < 0.05). This analysis identified 938 differential TADs between young and old and 1491 between young and geriatric (Figure 4I). In both comparisons, the majority of differences occurred in the left or right inter‐TAD regions, indicating that boundary insulation changes contribute more substantially to age‐associated 3D genome remodeling than alterations within domain interiors. Nevertheless, intra‐TAD differences were also observed, with approximately 20% of differential TADs showing loss of insulation in the old group and approximately 38% exhibiting loss of insulation in the geriatric group (Figure 4I, black stacked bars). Interestingly, we found several gene loci related to muscle stem cell function or myogenic potential were in differential TAD regions. The *Myf5*/*Myf6* locus showed a differential right inter‐TAD change in the young vs. geriatric comparison and also showed several differential loops in the old and geriatric samples (Figure 4J, left). These results may explain the increased expression of the *Myf5/Myf6* mRNAs in aged, particularly geriatric, MuSCs (Figure 2F). Another interesting change was at the *Spry1* locus, a gene known to regulate quiescence (Shea et al. 2010) (Figure 4J, right). A comprehensive list of TADs, differential TADs, TADs changed near known myogenic genes, loops, and differential loops is provided in Table S4.

Finally, to prioritize a list of enhancers likely to impact function, we implemented the Activity by Contact (ABC) model on the male MuSC data (Fulco et al. 2019). ABC integrates information from 3 omic layers: Activity (ATAC‐seq and H3K27ac CUT&RUN) and Contact (Hi‐C), to predict regulatory element activity (Figure S4I). To focus on enhancer‐gene pairs, we filtered out promoter and self‐promoter classes (self‐promoters are element‐gene pairs where the element is the promoter of that same gene) and aggregated ABC scores for young, old, and geriatric samples by summing the scores for each target gene to yield a single value per gene (*n* = 16,709). Following quantile normalization, we calculated the fold change (FC) in ABC scores in old and geriatric compared to young and performed a pathway enrichment analysis with target genes showing FC > 25. Enhancer‐linked genes showed strong enrichment of neuronal function‐related terms in the old vs. young comparison (Figure 4K, green). By contrast, the geriatric vs. young comparison revealed in addition to a few neuronal terms, pathways related to ECM remodeling and musculoskeletal differentiation (Figure 4K, blue). Additionally, inflammatory terms were modestly enriched in this comparison. Three example browser shot views of the *Sharpin* (inflammatory), *Unc13b* (neuronal) and *Nfatc1* (osteoblast differentiation) loci, with differential Hi‐C loops at 5 kb resolution, are shown in Figure S4J. Of note, most of these enhancer‐driven target genes did not prominently feature in our steady state bulk RNA‐seq measurements (Figure 2, see Section 3). Unlike inflammatory genes, collagen gene promoters showed no epigenetic changes despite decreased mRNA and protein levels with age (Figure 2C cluster 8, Figure 2F, Figures S2H, S3B, S3D, S4K) suggesting potential involvement of a post‐transcriptional regulatory mechanism.

Collectively, our results indicate that enhancer activity increases with age in MuSCs and may compromise cell identity and myogenic potential. Pending in vivo results, we speculate that targeting enhancers could be a viable strategy to overcome age‐associated decline in SKM function.

## 3 Discussion

MuSC dysfunction with age is a critical contributor to poor muscle regeneration, yet the molecular mechanisms remain incompletely understood. In this study, we conducted multi‐omic profiling of MuSCs across three age groups (young, old, and geriatric) and two sexes, in multiple biological replicates. The comprehensive dataset enabled us to validate both quantitative and qualitative trends in MuSCs during aging. We queried diverse “omic” layers including the epigenome (ATAC‐seq, CUT&RUN of H3K4me1 and H3K27ac), transcriptome (RNA‐seq), and the 3D genome (Hi‐C). Our results revealed temporal events including a loss of cell identity and a strong interferon response that initiated early in males but intensified in late life, and widespread 3D genome rewiring indicative of MuSC dysfunction.

While it is known that old muscles have fewer MuSCs, we found that male MuSCs exhibited lower basal proliferative capacity in the young group and further exacerbation in the old (Figure 1G,H). This functional difference was also reflected in the expression patterns of cell cycle‐related mRNAs (Figure 2B), early activation of nucleic acid sensors, and the interferon pathway (Figure 2D,E) in old males, as well as the elevated expression of ERVs in geriatric males (Figure 2G). ERVs can either employ viral mimicry to mount a strong inflammatory response via cGAS‐STING and NFκB, or activate the interferon response via RNA sensing (Di Giorgio and Xodo 2022). Ultimately, these antiviral mechanisms could reduce proliferative capacity. Although the reason for sex differences in the basal condition is unknown, it is possible that cell cycle or epigenetic regulators on the sex chromosomes could play a role. Several male‐specific proteins have been linked to NFκB interaction. For instance, Sex‐determining Region Y (SRY) has binding sites for NFκB in its 5′ region suggesting a potential direct interaction (Ross et al. 2008). We found strong expression of *Ddx3y*, which encodes an RNA helicase, in male MuSCs (Figure S2B), whereas it is typically expressed in the testis. DDX3X, the X chromosome counterpart, is known to activate type I interferon via regulation of TBK1 and IRF3 (Soulat et al. 2008), enhance p21 expression (Chao et al. 2006), and drive ER stress (Adjibade et al. 2017), all of which could decrease proliferative capacity.

One of the most interesting findings was that chromatin accessibility (in males) and enhancer‐associated histone modifications (in both sexes) show age‐dependent variations (Figures 3A and 4A, Figure S4C). The variation in chromatin accessibility (Figure 3A) and mRNA expression (Figure 2A,C) appears to be progressive, while enhancer marks (Figure 4A, Figure S4C) change more abruptly in the geriatric age group. However, even though most age‐associated transcriptomic alterations were progressive in both males and females (Figure 2C), we found a smaller subset of sex‐dimorphic inflammatory mRNAs that followed opposite trajectories in the two sexes (Figure 2H,I).

Three major patterns emerge from comparison across “omic” layers. First, transcriptomic data show a strong type I and II interferon response (Figure 2C–F, Figure S2D–F), partially due to activated ERV expression (Figure 2G). Concordantly, chromatin accessibility‐based TF activity measurements and IF show heightened NFκB activity, primarily at enhancers (Figure 3I–K). Finally, enhancer modifications, open chromatin, and 3D genome contacts reinforce inflammation (Figures 2C–F and 4K). Interestingly, classic senescence mRNA signatures in the SenMayo (Saul et al. 2022) or CellAge (Avelar et al. 2020) panels, did not seem to be particularly enriched (data not shown).

Second, beyond inflammation, the extensive enhancer rewiring and 3D genome reorganization also suggests a broader loss of myogenic identity and lineage drift (Figure 3E,F, Figure 4). Many of the chromatin changes occurred at non‐coding areas of the genome (Figures 3E,F and 4D, Figure S4F). Promoter regions that showed reduced accessibility with age were close to genes related to muscle function (Figure 3F,H). Furthermore, we found many genomic loci encoding key myogenic proteins were in differential TADs and showed altered loop formation (Figure 4I,J, Table S4). Integrated multi‐omic analysis identifying the top active enhancer ‐gene pairs revealed numerous neuronal terms in the old (Figure 4K, green). In the geriatric group, terms related to bone and cartilage development became evident (Figure 4K, blue), indicative of a shift towards a broader mesodermal commitment. Importantly, changes in enhancer activity do not correlate with bulk RNA‐seq data. We speculate that the altered multipotent cell state of geriatric MuSCs is purely epigenetic and is not reflected in the RNA due to the lack of differentiation signals along a specific lineage. Alternatively, nascent RNA‐seq methods such as GRO‐seq or PRO‐seq could better capture nascent transcription and identify instances of altered mRNA stability rather than steady‐state transcription.

Third, despite the extensive epigenome alterations (Figures 3 and 4, Figure S4), we found that the majority of collagen mRNAs were decreased with age (Figure 2C,F, Figure S2D–F), a trend that was also recapitulated at the protein level (Figure S2H). While the deterioration of the ECM is well‐documented in the SKM (Zhou et al. 2019), the large‐scale age‐dependent decrease of collagen expression in purified MuSCs has not been reported previously, likely because we constructed full‐length cDNA libraries using the SMART‐seq methodology which enables the capture of long transcripts. Collagen plays a crucial role in maintaining the quiescent state of MuSCs. The depletion of COLV disrupts the NOTCH‐COLV‐CALCR (calcitonin receptor) signaling cascade, leading to aberrant cell cycle entry and a gradual decline in the MuSC pool (Baghdadi et al. 2018). Concordantly, we observed decreased expression of *Pax7*, *Notch*, and *Calcr* with age (Figure 2F), indicating a loss of MuSC quiescence during aging. The *Spry1* locus, encoding an important mRNA known for quiescence maintenance (Shea et al. 2010), also showed 3D genome alterations (Figure 4J). Interestingly, we did not observe any change in chromatin accessibility or histone modifications at the promoters of collagen genes (Figure S4K), suggesting that collagen levels may be regulated post‐transcriptionally. In this regard, many RNA‐binding proteins, such as LARP6 and αCP (hnRNPE) (Stefanovic 2013), have been found to bind collagen mRNAs and influence their turnover rate. Similarly, a range of microRNAs (miRs) and long non‐coding RNAs (lncRNAs) associate with collagen mRNAs and regulate their turnover and post‐transcriptional fate (Wen et al. 2022). Notably, miR‐29a/b/c‐3p is known to target collagen mRNAs for degradation (Maurer et al. 2010), increase with age in rats (Hu, Klein, et al. 2014), and drive age‐related phenotypes (Swahari et al. 2024). Further studies are needed to fully elucidate how collagen mRNA turnover is regulated by these and other factors as a function of age.

In summary, our multi‐omic framework reveals temporally coordinated changes that converge on pathways linked to MuSC dysfunction with aging. We detect early, male‐biased activation of proinflammatory programs and in late life, extensive epigenomic remodeling consistent with lineage drift and loss of myogenic commitment. While deeper functional characterization of geriatric FISCs and direct evidence of age‐related cell fate changes are needed, our study provides insights into how multiple epigenetic programs collaborate during aging and nominate candidate targets for follow‐up functional studies to mitigate muscle decline.

## 4 Materials and Methods

### 4.1 Animals

This study was approved by the Animal Care and Use Committee of the NIA in Baltimore, MD under Animal Study Protocol number 481‐LGG‐2025 and 503‐LGG‐2028. Young, old and geriatric inbred C57BL6/JN mice of both sexes were acquired from the NIA aged rodent colony (https://ros.nia.nih.gov/) and housed in rooms that were maintained at 22.2°C ± 1°C and 30%–70% humidity. Routine tests are performed to ensure that mice are pathogen‐free and sentinel cages are maintained and tested according to American Association for Accreditation of Laboratory Animal Care (AAALAC) criteria. The age and sex information is available in Table S1.

### 4.2 Muscle Injury

Mice were anesthetized with 2% isoflurane by placing them inside an induction chamber for about 5 min. Mice were then removed from the chamber, covered with an anesthesia mask on a surgical bench, and the hair was removed from both lower hind limbs around the injection sites. Muscle injury was induced by injecting 50 μL of 1.2% barium chloride (Sigma) in 0.9% saline solution into approximately 25 sites in the lower hindlimb muscles (tibialis anterior [TA] and gastrocnemius).

### 4.3 Muscle Stem Cell Isolation

MuSCs were isolated by FACS as described previously (Liu et al. 2015). For muscle dissection, young, old and geriatric mice were euthanized with CO_2_ followed by cervical dislocation. After weighing the mouse, hindlimb muscles including TA, extensor digitorum longus (EDL), gastrocnemius, soleus, and quadriceps were collected, and visible fat and tendons were removed. All muscles from both hindlimbs were pooled, chopped with surgical scissors, and then transferred into 50 mL conical tubes. Minced muscles were digested with 1000 U/mL collagenase II (Worthington) and 1.5 U/mL dispase (Thermo Fisher) in a 37°C water bath with agitation (75 rpm) for a total of 1.5 h. Once the digestion was complete, mononucleated cells were further dissociated using a 10 mL syringe and a 20‐gauge needle by aspirating and ejecting the muscle suspension ten times. The cell suspension was then filtered using 40 μm nylon cell strainers. After centrifugation at 400 *g* for 5 min at room temperature (RT), the supernatant was discarded and the resulting cell pellets were immediately resuspended in 350 μL of staining solution with a cocktail of antibodies including CD31‐APC, CD45‐APC, SCA1–Pacific Blue, and VCAM1‐biotin (Biolegend), and then incubated at 4°C for 45 min in the dark. Sample volumes were adjusted to 2 mL with staining solution minus antibodies, centrifuged at 400 *g* for 5 min at 4°C, and the supernatant was discarded. Pellets were then resuspended in a staining solution containing PECy7: Streptavidin (1:100; Biolegend) and 0.3 μg/mL PI (Thermo Fisher) and incubated for another 15 min at 4°C in the dark. After incubation, sample volumes were adjusted to 2 mL with wash medium (Ham's F‐10 [Cytiva] supplemented with 10% horse serum [Thermo Fisher] and 1× penicillin/streptomycin [Thermo Fisher]), centrifuged at 400 *g* for 5 min at 4°C, the supernatant was discarded, the pellet was re‐suspended in 2 mL wash medium, and then filtered through 35 μm cell strainers. Aria Fusion sorter (BD Biosciences) was used to isolate MuSCs using VCAM1 as a positive marker, and propidium iodide (PI), CD31, CD45, and SCA1 as negative markers. The purity of the isolated MuSCs was assessed by taking a small number of cells followed by immunofluorescent staining of the MuSC marker, PAX7 (see Section 4.5 for details).

### 4.4 In Vitro Muscle Stem Cell Expansion and Differentiation

Prior to MuSC seeding, 8‐well chamber slides (Ibidi) were coated with 0.1 mg/mL poly‐D‐lysine solution (Sigma) at RT for ~8 h followed by ECM gel (Sigma, 1:100) at 4°C for at least 6 h. FISCs from young or old mice were seeded in the coated 8‐well chamber slides at 5000 cells/well. FISCs were cultured in GM (40% Ham's F‐10 medium [Cytiva], 40% DMEM [Thermo Fisher], 20% FBS [Thermo Fisher], and 1% penicillin/streptomycin [Thermo Fisher]) supplemented with 5 ng/mL hFGF (Sigma) for 5–7 days. The GM with freshly added hFGF was changed every 2 days. Wells containing myogenic progenitors during proliferation were counted by brightfield microscopy or fixed with 4% paraformaldehyde (PFA) and stained with Hoechst; the number of cells per well was counted as Hoechst‐stained nuclei at the indicated time points. After 6–8 days of proliferation in GM, the media were changed to DM (98% DMEM [Thermo Fisher], 2% horse serum [Thermo Fisher], and 1% penicillin–streptomycin [Thermo Fisher]) and cultured for an additional 3–4 days during which time myotubes were formed. Myotubes were immuno‐stained with myosin heavy chain (MHC) and DAPI. The fusion index of cells was calculated as the percentage of nuclei (DAPI) in myotubes to all nuclei visible in the field.

### 4.5 Immunofluorescence

FISCs (~5000 cells/well) were seeded in an 8‐well chamber slide (Ibidi) coated with poly‐D‐lysine (Sigma) and ECM gel (Sigma) for ~8 h. FISCs were fixed with 4% PFA for 5 min at RT. After washing with PBS, cells were permeabilized with 0.3% Triton X‐100 in PBS and blocked in 5% BSA in PBS for 30 min at RT. To confirm the purity of FISCs isolated by FACS, cells were incubated with anti‐PAX7‐s antibody (1:50, Developmental Studies Hybridoma Bank) in 2.5% BSA solution overnight at 4°C in a humidified chamber. For myotube quantification, cells were incubated with anti‐MYH3 antibody (1:100; Santa Cruz Biotechnology); for collagen staining, cells were incubated with anti‐collagen I (1:100; Abcam) or anti‐collagen III (1:100; Abcam) antibodies; and for NFκB staining, cells were incubated with phospho‐p65 (1:500; Cell Signaling Technologies) antibody. The following day, cells were washed four times with PBS with 0.1% Tween‐20 (PBST) and incubated with secondary antibodies conjugated to a fluorescent dye (1:1000, Alexa Fluor 488 or Alexa Fluor 647) for 1 h at RT followed by washes with PBST. Finally, cells were incubated with 1 μg/mL DAPI in PBS to stain nuclei for 5 min at RT. Images were captured using a Zeiss LSM 710 confocal microscope and subsequently split into different channels. Intensities were measured overlapping DAPI using Image J (https://imagej.nih.gov/ij/).

### 4.6 Cytokine Array

FISCs (~150,000 per sample) were seeded in each well of a chamber slide (Ibidi) that had been treated with 0.1 mg/mL poly‐D‐lysine (Sigma) and 100 μg/mL of freshly prepared ECM gel (Sigma). The cells were cultured in serum‐free DMEM for 24 h. Cell culture supernatants were collected, centrifuged, and transferred to a new tube. After filtration through 0.22 μm filters, the supernatant was dried in a speed‐vac until it became a powder, then resuspended in PBS and stored at −80°C. The multiplex analysis was performed using the Luminex 200 system (Luminex) by Eve Technologies Corp. (Calgary, Alberta). Forty‐five cytokines were simultaneously measured in the samples using the Mouse Cytokine 45‐Plex Discovery Assay (Eve Technologies).

### 4.7 RNA Extraction and RNA‐Seq Library Preparation

Sorted cells were centrifuged at 2500 *g* for 5 min at 4°C and stored in −80°C. RNA was extracted from FISCs (~200,000 cells per sample) using the RNeasy Plus Micro Kit (QIAGEN) according to the manufacturer's instructions. Reverse transcription was performed with 1–10 ng RNA using the SMART‐Seq v4 Ultra Low Input RNA Kit (Takara) according to the manufacturer's instructions. After purification of amplified cDNA, quality and quantity were determined using a 2100 Bioanalyzer the DNA high sensitivity kit (HS, Agilent). RNAseq library constructions were performed using the SMART‐Seq Library Prep Kit (Takara) according to the manufacturer's instructions. Equimolar amounts of each library were combined, and the pooled library was further quantified using a NEBNext Library Quant Kit (New England Biolabs). The RNA‐seq libraries were subjected to two rounds of 100 bp paired‐end sequencing on a Novaseq 6000 platform using a SP 200‐cycle kit (Illumina).

### 4.8 ATAC‐Seq

ATAC‐seq was performed as described previously (Grandi et al. 2022). Briefly, 1 mL of cold ATAC‐seq wash buffer was added to thaw cryopreserved FISCs (~60,000 cells per sample) in BAM Banker Cryopreservative (Fisher Scientific), and pelleted by centrifugation at 500 *g* for 5 min at 4°C. The pellet was resuspended in 50 μL of ice‐cold ATAC‐seq lysis buffer (10 mM Tris‐Cl [pH 7.5], 10 mM NaCl, 3 mM MgCl_2_, 0.1% NP‐40, 0.1% Tween 20, and 0.01% digitonin) and incubated on ice for 3 min. The cell lysate was mixed with 1 mL of ATAC‐seq wash buffer (10 mM Tris‐Cl [pH 7.5], 10 mM NaCl, 3 mM MgCl_2_, and 0.1% Tween 20) followed by centrifugation at 500 *g* for 10 min at 4°C. The cell pellet was resuspended in transposition mix including 2.5 μL of Tagment DNA enzyme 1 (Illumina), 0.1% Tween 20, and 0.01% digitonin and incubated at 37°C for 30 min on a thermomixer (Eppendorf) at 1000 rpm. The transposed DNA fragments were PCR‐amplified using NEBNext Ultra II Q5 2× Master Mix (New England Biolabs). The sample was purified using the DNA Clean and Concentrator‐5 Kit (Zymo). The library quality and quantity were verified using the high sensitivity DNA kit on a 2100 Bioanalyzer (Agilent) and qPCR with NEBNext Library Quant kit (New England Biolabs), respectively. The ATAC libraries were subjected to 50 bp paired‐end sequencing on a NextSeq 2000 platform using a P2 100‐cycle kit (Illumina).

### 4.9 CUT&RUN

CUT&RUN was performed to profile enhancer histone modifications H3K4me1 and H3K27ac from young, old and geriatric FISCs following guidelines in the CUTANA CUT&RUN Protocol (EpiCypher). Briefly, FISCs were pelleted at 600 *g* for 5 min at 4°C and resuspended in wash buffer (20 mM HEPES‐KOH, pH 7.9, 150 mM NaCl, 0.5 mM spermidine, freshly added protease inhibitors (Roche), 1 mM PMSF, and 1 mM sodium butyrate). ~10,000 cells per sample were bound to activated Concanavalin A beads (Bangs Laboratories). The bead‐bound cells were incubated with antibody in wash buffer supplemented with 2 mM EDTA and 0.01% digitonin overnight at 4°C; digitonin was added to permeabilize cells for antibody binding in situ. After washing with 0.01% digitonin in wash buffer, CUTANA pAG‐MNase (1:20) was added to each sample and incubated at RT for 10 min to allow binding to antibody‐labeled chromatin. Samples were then equilibrated in ice water for 5 min. 2 mM CaCl_2_ was added to each sample to activate MNase and cleave antibody‐bound chromatin. After 2 h of incubation with CaCl_2_ at 4°C, reactions were stopped by adding 33 μL stop buffer (340 mM NaCl, 20 mM EDTA, 4 mM EGTA, 50 μg/mL RNaseA, 50 μg/mL glycogen) to degrade RNA and release DNA fragments. DNA was purified using the Monarch DNA Cleanup Kit (New England Biolabs) following the manufacturer's instructions. The concentration of CUT&RUN‐enriched DNA was determined using the Qubit fluorometer (Thermo Fisher) with a DNA HS kit (Agilent). Purified DNA (~2 ng) was used to prepare libraries using the NEBNext Ultra II library preparation kit with unique dual index PCR primers (New England Biolabs). The library concentrations were determined using the Qubit fluorometer with a DNA HS kit. Library quality and quantity were confirmed on a BioAnalyzer (Agilent) high sensitivity DNA chip. Equimolar amounts of each library were combined, and the pooled library was further quantified using a NEBNext Library Quant Kit (New England Biolabs). The CUT&RUN libraries were subjected to paired‐end sequencing with read lengths of 50 bp on a NextSeq 2000 platform using a P2 100‐cycle kit.

### 4.10 Hi‐C

Hi‐C of FISCs was performed with a modified low cell number protocol using the Arima‐HiC kit (Arima Genomics). Approximately 800,000 FISCs per sample (pooled from 3 to 4 male animals, Table S1) were cross‐linked with 2% formaldehyde for 10 min at RT and quenched with 0.2 M Glycine. Cell lysis, chromatin digestion, biotin labeling, and proximity ligation were performed following kit instructions with slight modifications for low cell number. Proximally ligated DNA was purified using 0.45 X SPRI beads (Beckman Coulter) and fragmented using a Covaris S220 instrument (peak incident power 140 W, duty cycle 10%, cycles per burst 200, time 95 s) to obtain an average fragment size of ~400 bp. Fragment size distribution was determined on a Bioanalyzer using a DNA HS kit (Agilent). After purification using SPRI beads (Beckman Coulter), biotinylated fragments containing ligation junctions were enriched using biotin enrichment beads. Hi‐C library was prepared on‐bead using the Accel‐NGS 2S Plus kit (Swift Biosciences). After adaptor ligation, ligated products were PCR‐amplified using KAPA Library Amplification Kit (Roche); the number of PCR cycles was determined using KAPA Library Quantification kit (Roche) before PCR. Finally, amplified libraries were purified using 0.9 X SPRI beads (Beckman Coulter). A shallow sequencing (75 bp paired end) of 4 million reads per library was performed on a MiniSeq platform (Illumina) to estimate library complexity. Libraries were combined, and the pooled library was quantified by qPCR using a NEBNext Library Quant Kit (New England Biolabs). The final pooled library was paired‐end sequenced with read lengths of 150 bp on a NovaSeq 6000 platform (Illumina) using an S2 300‐cycle kit.

### 4.11 Bioinformatic Analysis

#### 4.11.1 RNA‐Seq Analysis

Illumina sequencing reads (~32 million paired‐end reads per sample) were de‐multiplexed using bcl2fastq/2.20.0. Reads were trimmed to remove adapter sequences using trimmomatic/0.39 (Bolger et al. 2014). The quality of the resulting FASTQs was assessed using FastQC/0.11.9 (Andrews 2017) and MultiQC/1.9 (Ewels et al. 2016). Reads were aligned to the mouse reference genome (assembly GRCm38/mm10) using STAR/2.7.5b (Dobin et al. 2013). BAM files were sorted and indexed using samtools/1.10 (Li et al. 2009), and duplicates were removed using Picard/2.20.8. The BAM files were then filtered to retain alignments with a minimum mapping quality of 10 using samtools/1.10 (Li et al. 2009). The featureCounts function of the Rsubread R package/2.6.4 (Liao et al. 2019) was used to estimate counts. Differential gene expression analysis to uncover age‐altered expression was performed with the R Bioconductor package, DESeq2/1.30.1 (Love et al. 2014). Separately, differential expression analysis was performed using DESeq2/1.46.0 with a design including age and sex, followed by variance‐stabilizing transformation of significant genes. Genes were clustered by *k*‐means (*k* = 4) to identify expression patterns, and cluster‐specific enrichment was assessed using clusterProfiler/4.14.6. For TE expression, trimmed FASTQs were aligned allowing for multimapping (‐‐outFilterMultimapNmax 100 and ‐‐winAnchorMultimapNmax 100) using STAR/2.7.10b (Dobin et al. 2013) with unsorted BAM files as output. Significantly altered transcripts were assessed with the TEtranscripts function (‐‐mode multi) in TEToolkit/2.2.3 (Jin and Hammell 2018). Volcano plots, heatmaps and bubble plots were created using the R Bioconductor packages ggplot2 and pheatmap in R/4.0.

#### 4.11.2 ATAC‐Seq Analysis

ATAC‐seq data were processed by the Encyclopedia of DNA Elements (ENCODE) pipeline (https://www.encodeproject.org/atac‐seq/). Briefly, raw sequencing reads were trimmed to remove adapters and low‐quality bases using Cutadapt/1.9.1 and assessed for quality using FASTQC/0.11.9 (Andrews 2017), followed by alignment to the reference genome (GRCm38/mm10) using Bowtie/2‐2.2.9 (Langmead and Salzberg 2012). Mapped reads were then filtered to remove low‐quality and duplicate reads using Picard/2.10.6. deepTools/3.5.0 (Ramirez et al. 2014) bamCoverage function was used to convert BAM to bigWigs. Peak calling is performed using MACS2/2.1.1 to identify regions of open chromatin. DiffBind/3.2.6 (Stark and Brown 2011) was used to generate a PCA plot and identify differentially accessible regions between the samples. AUC values were generated using bwtool/1.0 (Pohl and Beato 2014). Enriched TF motifs were identified using the findMotifsGenome function of HOMER/5.1 (http://homer.ucsd.edu/homer/ngs/peakMotifs.html) with ATAC consensus peaks with 50% overlap between young and geriatric samples as background, and the ‐‐size parameter at 50 bp. TF footprinting analysis was performed using TOBIAS (Bentsen et al. 2020). TF binding site prediction and differential analysis were performed with TOBIAS's BindDetect function.

#### 4.11.3 CUT&RUN Analysis

CUT&RUN analysis was performed as outlined in Zheng et al., following the protocols.io tutorial (https://doi.org/10.17504/protocols.io.bjk2kkye). Briefly, sequencing reads (~7 million paired‐end reads per sample) were de‐multiplexed to generate compressed FASTQ files by the DRAGEN informatics pipeline on NextSeq 2000. Paired‐end reads were trimmed using Trim Galore/0.6.6 to remove the adapter. The qualities of the FASTQs were assessed using FASTQC/0.11.9 (Andrews 2017) and MultiQC/1.10 (Ewels et al. 2016). Reads were then aligned to the mouse genome (mm10) using Bowtie2/2.4.2 (Langmead and Salzberg 2012) with the parameters ‐very‐sensitive‐local, ‐I 10, and ‐x 700. Sam output files from Bowtie2 were then filtered to retain alignments with a minimum mapping quality of 2 using samtools/1.9 (Li et al. 2009). Aligned reads mapping to the Encyclopedia of DNA Elements (ENCODE) blacklist regions were removed using the intersect function in bedtools/2.30.0 (Quinlan and Hall 2010). RPKM (reads per kilobase per million mapped reads) normalized bigWig files were generated using the bamCoverage function in deepTools/3.5.0 (Ramirez et al. 2014). Sorted bam files were used to call broad peaks using MACS/2.2.7.1 (Zhang et al. 2008); the *q* value was set at 0.05. Differential peak analysis was performed using DiffBind/3.2.6 (Stark and Brown 2011). ROSE (Rank Ordering of Super‐Enhancers) (Whyte et al. 2013) algorithm was used to classify enhancers. AUC values were generated using bwtool/1.0 (Pohl and Beato 2014).

#### 4.11.4 Hi‐C Analysis

Illumina sequencing reads (~1.14 billion paired‐end reads per sample) were de‐multiplexed using bcl2fastq/2.20.0. Individual FASTQ files from each age group were merged and then aligned to the assembly GRCm38/mm10 using Burrows‐Wheeler Aligner (bwa/0.7.17) to generate SAM files. Subsequently, pairtools/1.0.2 was used to create .pairsam, which was sorted and deduplicated before generating .pairs and BAM files. The BAM files were sorted and indexed using samtools/1.19 (Li et al. 2009) before being further processed using juicer_tools pre option (juicer/1.6; Durand et al. 2016) to make .hic files at multiple resolutions with MAPQ scores ≥ 30 and Arima provided fragment‐delimited maps. Hi‐C matrices from young, old, and geriatric samples were depth‐normalized to the smallest library using hicNormalize (hicExplorer/3.7.6). TADs were called with hicFindTADs at an FDR threshold of 0.001 in each age group. For differential analysis, the young TADs (*n* = 2261) were used as the reference in hicDifferentialTAD and evaluated for differences across intra‐TAD, left‐inter‐TAD, and right‐inter‐TAD regions. A TAD was considered differential if at least one of these regions showed a statistically significant change (*p* < 0.05). Differential TADs were annotated using annotatr/1.24.0. Differential loops were called at 5 kb resolution using KR normalization with mustache's diffmustache.py package in python/3.1 (detection FDR < 0.05 and difference FDR < 0.05) (Roayaei Ardakany et al. 2020).

#### 4.11.5 ChromHMM

Multiple chromatin datasets (binarized bam files) including ATAC‐seq (this study), CUT&RUN for histone modifications H3K4me1, H3K27ac (this study) and published ChIP‐seq for H3K4me3 and H3K27me3 (Liu et al. 2013) were integrated to predict chromatin states using the LearnModel command in ChromHMM (Ernst and Kellis 2017), where the number of states was set to 10.

#### 4.11.6 Activity by Contact (ABC) Model

The ABC model was implemented in python/3.1.0 by running the snakemake pipeline described by Fulco et al. (2019) using default rule parameters. ATAC‐seq tagAlign files were used as the accessibility feature, H3K27ac CUT&RUN signal as a proxy for enhancer activity and merged .hic files as contact maps. After filtering out self‐promoters and rows annotated with a “promoter” class from the ABC output, ABC scores for young, old, and geriatric samples were aggregated by summing the scores for each target gene to yield a single value per gene. These values were then merged into a single matrix, which was quantile normalized using the normalizeBetweenArrays function from the limma/3.60.6 R package (Ritchie et al. 2015). A fold change of > 25 was then used to compute the enrichment of biological pathways in old vs. young or geriatric vs. young comparisons using the enrichGO function in clusterProfiler/4.14.3 (Yu et al. 2012) in R/4.4.1.

#### 4.11.7 PCA Plots

RNA‐seq PCA plot was generated in R/4.0 with the DESeq2 output and plotted with ggplot2. ATAC‐seq and CUT&RUN peak PCA was generated with DiffBind (Stark and Brown 2011). Hi‐C PCA plots were generated in R/4.4.1 using outputs from FAN‐C's (Kruse et al. 2020) pca function.

#### 4.11.8 Correlation Plots

Correlation analysis for RNA‐seq data were performed in R/4.4.1 using the DESeq2/1.46.0 and pheatmap/1.0.13 packages. Variance‐stabilized counts were used to compute Spearman's rank correlations between samples, and correlation heatmaps were generated to assess replicate clustering. For ATAC‐seq or CUT&RUN, bigWig files containing signal tracks were processed using the rtracklayer and BSgenome.Mmusculus.UCSC.mm10 packages in R. The mouse genome was tiled into 10 kb bins, and the average signal intensity per bin was quantified across all samples. Bins with nonzero coverage were retained, and pairwise Spearman correlation coefficients were computed to assess similarity between samples. The resulting correlation matrix was visualized as a clustered heatmap using the pheatmap package. Spearman correlation between Hi‐C matrices were performed using hicExplorer/3.7.6 hicCorrelate at 1 mb.

#### 4.11.9 Heatmaps

Signal intensity across TSSs and enhancer regions was calculated using the computeMatrix function and a heatmap was generated with the plotHeatmap function in deepTools/3.5.0 (Ramirez et al. 2014).

#### 4.11.10 Area Under the Curve (AUC) Calculation

bwtool/1.0 (Pohl and Beato 2014) was used to get genome coverage information (AUC) across regions of interest and then plotted with ggplot2 in R/4.0.

#### 4.11.11 Annotation

Genomics regions were annotated using the ChIPseeker/1.26 annotatePeak function in R/4.0 (Yu et al. 2015).

#### 4.11.12 Gene Ontology and Gene Set Enrichment Analysis

GO analysis (Huang da et al. 2009) of the DARs was performed using DAVID/6.8 with *Mus musculus* genes as background. The top 10 significant GO terms for the biological process category sorted by fold enrichment are reported. Age‐related pathways were identified using GSEA/v4.3.2 (Mootha et al. 2003), following the guidelines provided in the software documentation. A total of *n* = 11,649 genes were ranked based on their differential expression changes and statistical significance. The GO biological processes database was used as the reference gene set. To calculate *p*‐values for each pathway, 1000 random permutations were performed. The top 10 significant age‐related pathways (FDR < 0.01 for old vs. young and geriatric vs. young, and FDR < 0.1 for geriatric vs. old) for each comparison were visualized in bubble plots, using normalized enrichment scores (NES) to represent pathway enrichment.

#### 4.11.13 Genome Browser Tracks

Genome browser tracks were created for merged (across replicates) samples by converting the BAM files to bigWig files using the bamCoverage function of deepTools/3.5.0 (Ramirez et al. 2014) then uploaded on the University of California, Santa Cruz Genome Browser using custom tracks.

## Author Contributions

P.S. conceptualized the project. C.S. and P.S. wrote the manuscript. C.S. performed all wet lab experiments with help from N.Y., E.P., L.W., C.‐Y.C., L.L. and T.R. provided important tips for MuSC isolation and low‐input RNA‐seq. V.S. lab assisted in the optimization of muscle stem cell isolation. C.D. performed FACS for muscle stem cell isolation. N.Y., J.R.O., F.B., and J.C. assisted in Hi‐C analysis. Q.M., and N.B. provided important input into CUT&RUN analysis. J.R.O., C.S., N.Y., and P.S. performed all bioinformatics analyses. J.‐H.Y. helped with MHC staining. J.F. and S.D. provided sequencing support.

## Disclosure

Quantification and Statistical Analysis: Statistical analyses for all experiments were performed in R or GraphPad Prism/9.0.0. Statistical data are presented as mean ± SEM. Sample size (*n*), statistical tests used, and *p*‐values are specified in the figure legends.

## Conflicts of Interest

The authors declare no conflicts of interest.

## Supporting information


**Figure S1:** MuSC isolation by FACS and purity verification (related to Figure 1).
**Figure S2:** Verification of transcriptomic changes during MuSC aging (related to Figure 2).
**Figure S3:** Verification of transcriptomic and chromatin accessibility changes during MuSC aging (related to Figures 2 and 3).
**Figure S4:** Alteration of H3K4me1 and 3D genome during MuSC aging (related to Figure 4).

**Table S1:** Information on animals used in this study.

**Table S2:** Differential mRNAs from RNA‐seq data.

**Table S3:** Differential peaks from ATAC‐seq data.

**Table S4:** Differential peaks, TADs, and loops from CUT&RUN and Hi‐C data.

## Data Availability

The data that support the findings of this study are openly available in Gene Expression Omnibus at https://www.ncbi.nlm.nih.gov/geo/, reference number GEO: GSE293139. Source data are available at MendeleyData (DOI: 10.17632/9hzf5by49f.1). All original code has been deposited at https://github.com/PSenlab/Shi_2025 and is publicly available.
