# Supplementary material for: Enhancer Rewiring Orchestrates Inflammation and Loss of Cell Identity During Muscle Stem Cell Aging
Source: Aging Cell. 2025 Nov 14;25(1):e70289. doi: 10.1111/acel.70289 (PMC12740102; doi:10.1111/acel.70289)
Supplement: Supplementary file 1 — Figure S1: MuSC isolation by FACS and purity verification (related to Figure 1). Figure S2: Verification of transcriptomic changes during MuSC aging (related to Figure 2). Figure S3: Verification of transcriptomic and chromatin accessibility changes during MuSC aging (related to Figures 2 and 3). Figure S4: Alteration of H3K4me1 and 3D genome during MuSC aging (related to Figure 4). [file ACEL-25-e70289-s005.docx]

**Supplementary Figures**

**Supp. Figure 1**


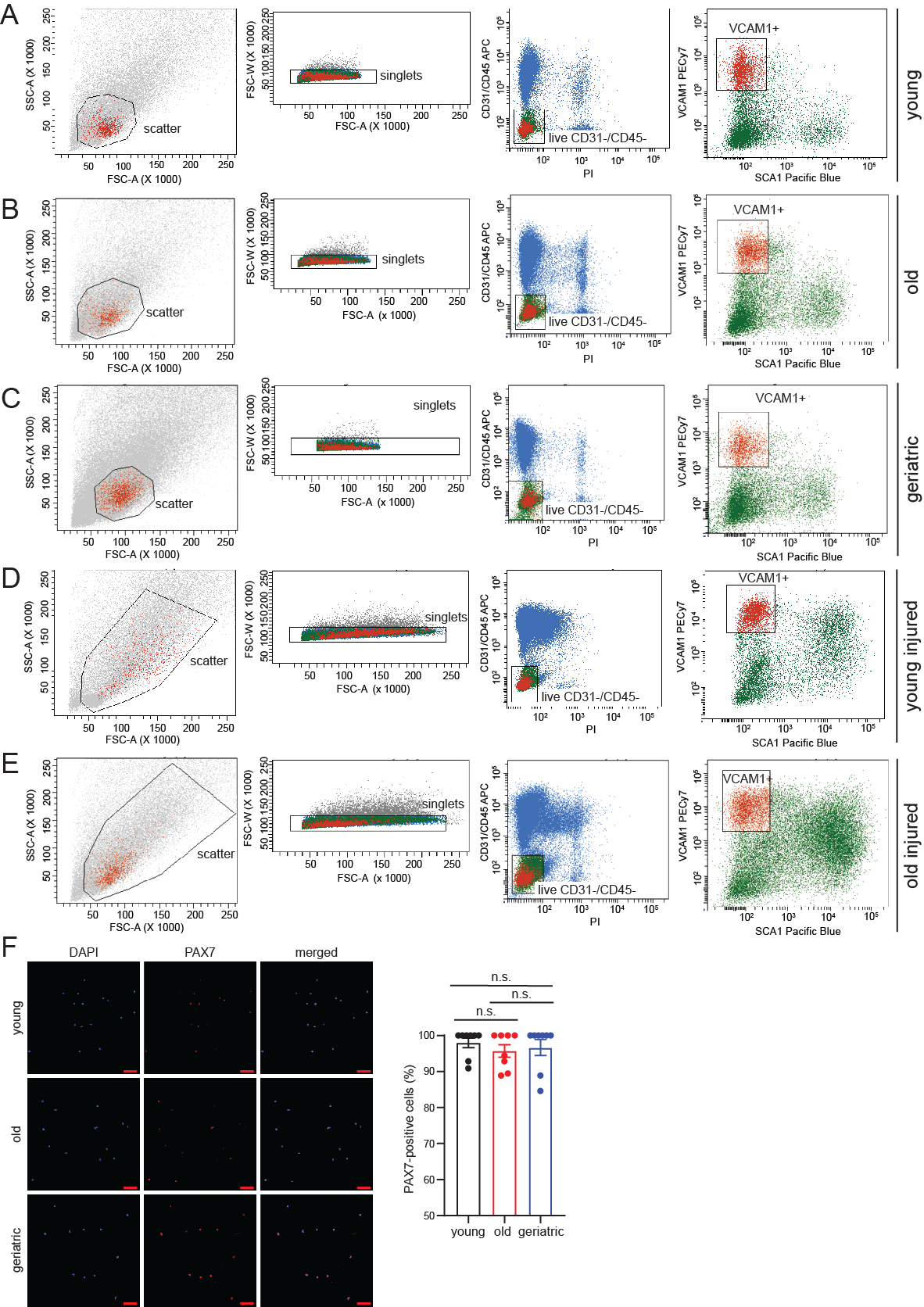


**Supp. Figure 1. MuSC isolation by FACS and purity verification (related to Figure 1)**

**(A)** Representative FACS plots showing profiles of young FISCs from uninjured muscles. **(B)** Same as (A) except for old FISCs. **(C)** Same as (A) except for geriatric FISCs. **(D)** Representative FACS plots showing profiles of young FISCs from injured muscles. **(E)** Same as (D) except for old FISCs. For A-E, before sorting, a gate on the FSC-A and SSC plot was created to exclude debris, followed by another gate on the FSC-W and FSC-A plot to collect singlets. Two consecutive gates were then used to collect the viable VCAM^+^CD31^−^CD45^−^SCA1^−^ population, VCAM1^+^ cells (red) in the rightmost plots are MuSCs. **(F)** IF of FISCs, ~8 hours after plating showing PAX7 (red) and DAPI (blue), the scale bar represents 50 μm. The bar plot shows the purity (PAX7^+^ cells) of FISCs from young, old, and geriatric mice, data represented as mean ± SEM, n=8 images per replicate. Significance was calculated using an unpaired two-tailed Welch’s t-test and revealed no statistically significant differences. For panels (A-F), only male mice data is shown.

**Supp. Figure 2**


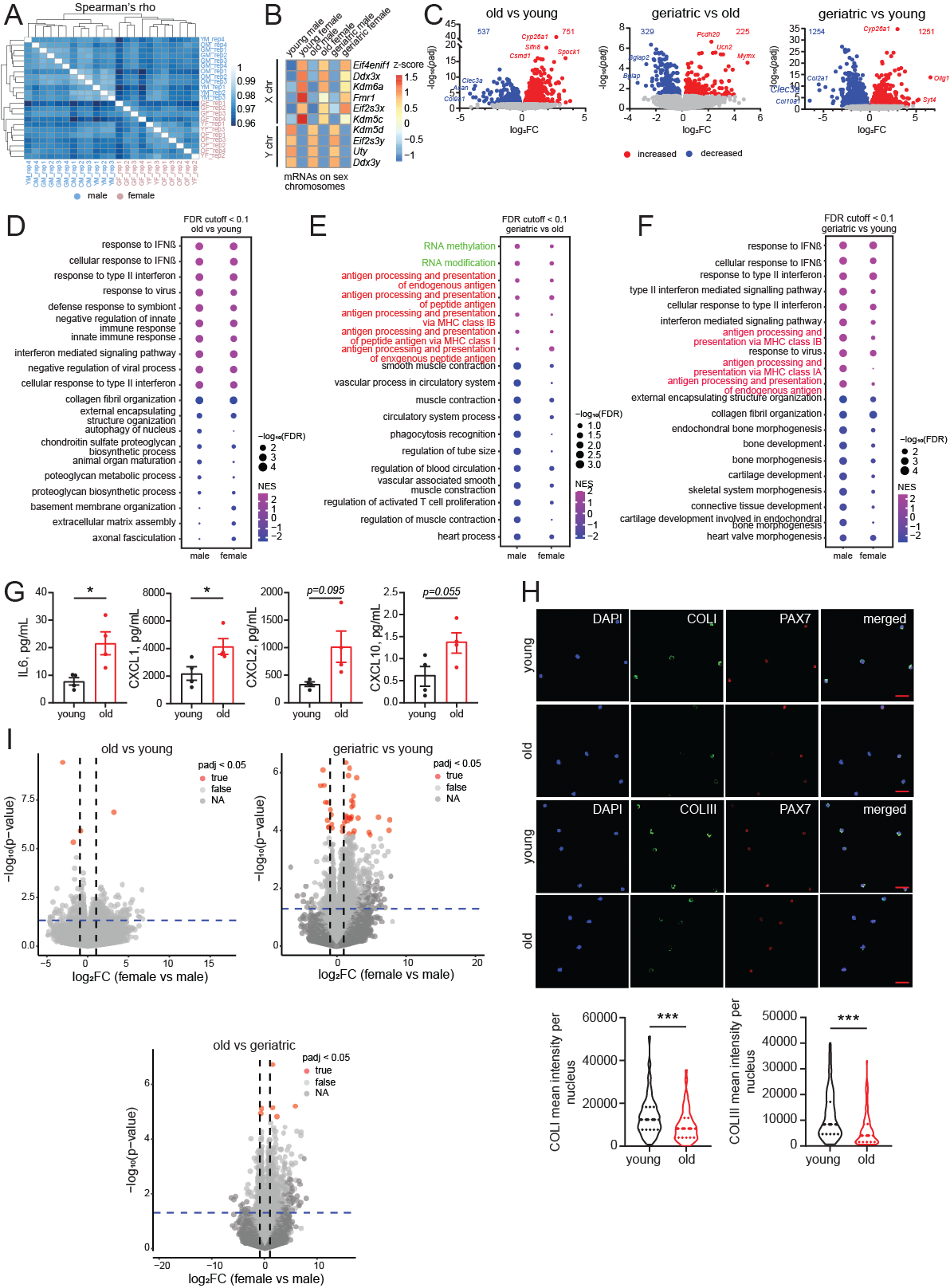


**Supp. Figure 2: Verification of transcriptomic changes during MuSC aging (related to Figure 2)**

**(A)** Heatmap showing Spearman correlation coefficients between RNA-seq samples, clustered by correlation distance. **(B)** Heatmap of mRNAs transcribed from genes located on the sex chromosomes. **(C)** Volcano plots of DARs in FISCs, comparing old vs young (left), geriatric vs old (middle), and geriatric vs young (right). Blue and red dots on the left and right indicate significantly increased and decreased DARs, respectively (FDR < 0.05). **(D)** Bubble plot showing the top 10 significant (FDR < 0.1) enriched or de-enriched pathways in old vs young for both male and female. Color scale shows normalized enrichment scores (NES) from GSEA. The bubble size indicates -log_10_ FDR q-values. **(E)** Same as (D) except in geriatric vs old. **(F)** Same as (D) except in geriatric vs young. For (E-F), pathways related to antigen processing and presentation are labeled in red while those related to RNA modification are in green. **(G)** Multiplexed LASER bead assay of cytokine levels in conditioned medium from young and old FISCs cultured for 36 h. Data are presented as mean ± SEM from n=4 mice in each group. * p < 0.05 by an unpaired two-tailed Welch’s t-test. **(H)** IF images of COLI and COLIII in young and old FISCs with mean signal intensity per cell quantifications on the bottom. Scale bar represents 20 μm. *** p < 0.001 by an unpaired Welch’s t-test. **(I)** Volcano plots for sex-dimorphic gene expression across age groups, with significantly differentially expressed genes (adjusted p < 0.05) highlighted in red. For panels (A-F and I), both males and females were considered, for (G-H) only male was used.

**Supp. Figure 3**


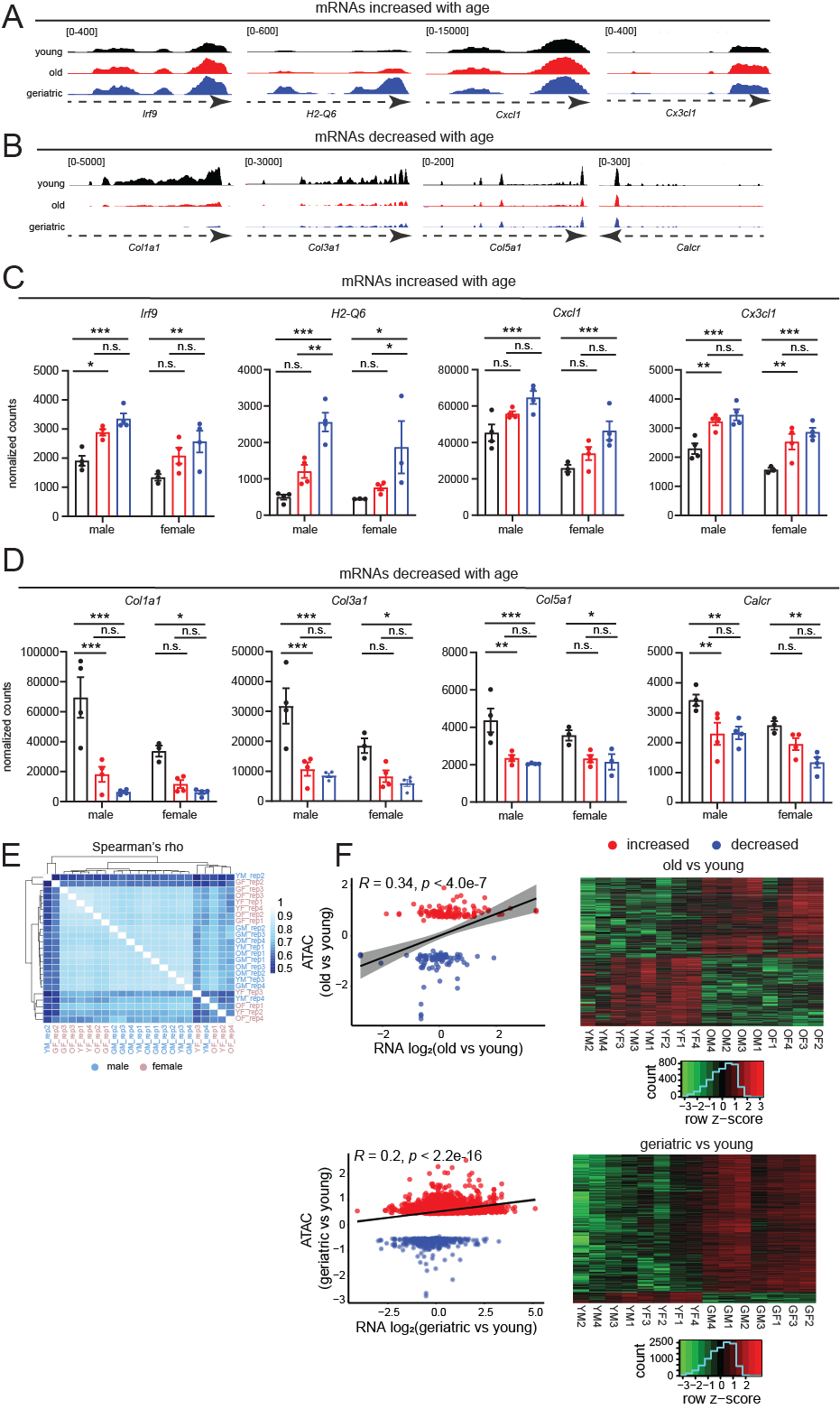


**Supp. Figure 3: Verification of transcriptomic and chromatin accessibility changes during MuSC aging (related to Figures 2 and 3)**

**(A)** Genome browser snapshots showing RNA-seq signal across genes encoding mRNAs increased with age in young, old, and geriatric FISCs. **(B)** Same as (A) except for mRNAs decreased with age. **(C)** Bar graph showing DESeq2-normalized counts of mRNAs increased with age for both males and females (data represented as mean ± SEM, n=4). **(D)** Same as (C) except for mRNAs decreased with age. For (C-D), significance was calculated using two-way ANOVA with Tukey’s multiple comparisons test: * p < 0.05, ** p < 0.01, *** p < 0.001. **(E)** Heatmap showing Spearman correlation coefficients of ATAC-seq signal across 10 kb genomic bins, clustered by correlation distance. **(F)** Scatterplots showing correlation between ATAC peak signal and RNA expression change in old vs young (top) and geriatric vs young (bottom). Each dot represents an mRNA identified as differentially abundant by DESeq2. Heatmaps on the right of the scatterplots show affinities for differential ATAC peaks. Row z-score color scale is shown below. For panels (A-F), both males and females were considered.

**Supp. Figure 4**


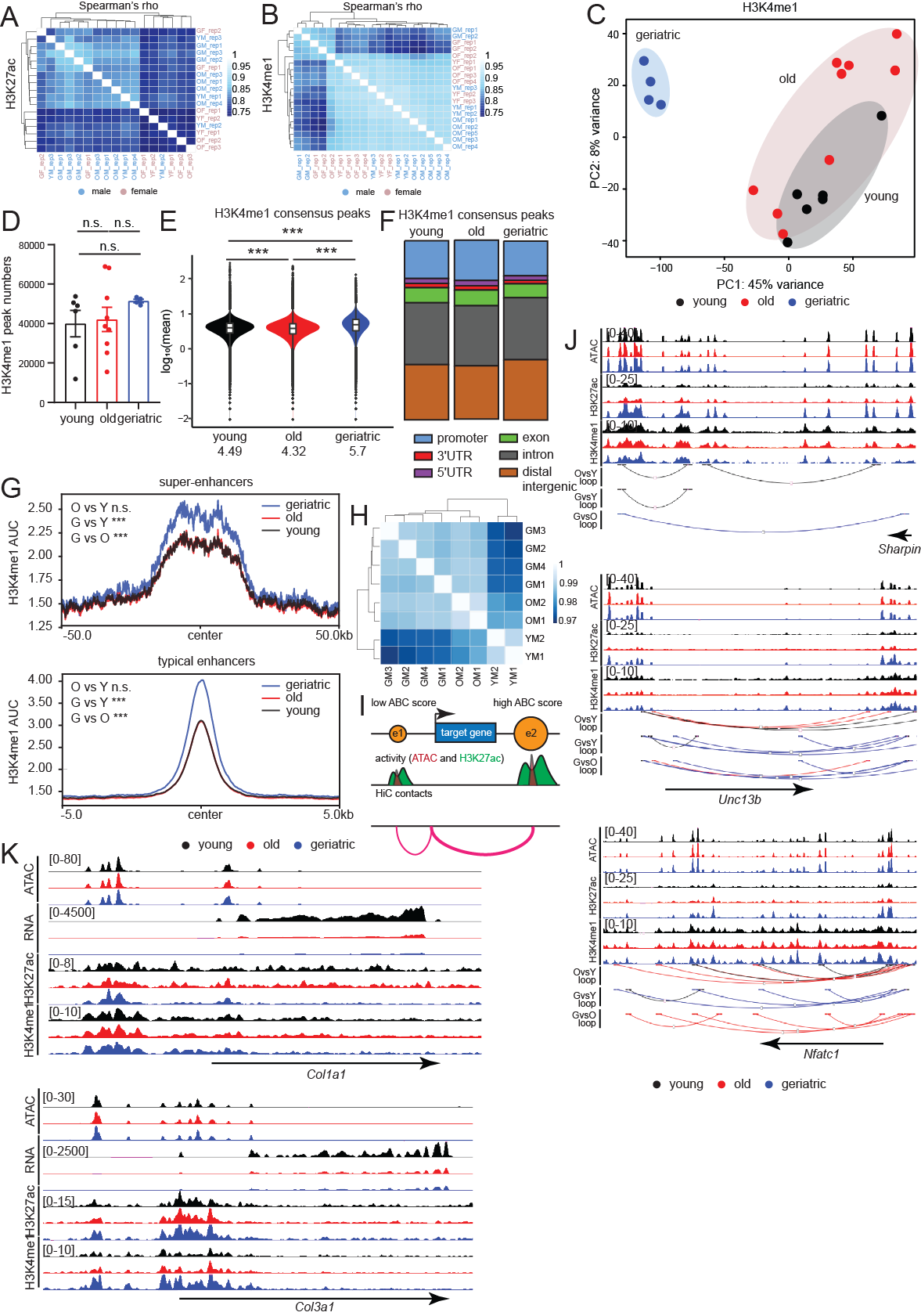


**Supp. Figure 4: Alteration of H3K4me1 and 3D genome during MuSC aging (related to Figure 4)**

**(A)** Heatmap showing Spearman correlation coefficients of H3K27ac CUT&RUN signal, clustered by correlation distance. **(B)** Same as (A) except for H3K4me1. **(C)** PCA plot of H3K4me1 data from young, old, and geriatric FISCs. **(D)** Barplot showing the number of H3K4me1 peaks in young, old, and geriatric FISCs, data represented as mean ± SEM, n=4-9 mice. Significance was calculated using one-way ANOVA with Tukey’s multiple comparisons test and revealed no statistically significant differences. **(E)** Violin plot showing the distribution of mean intensity of consensus H3K4me1 peaks in young, old, and geriatric FISCs. Median values are indicated below. *** p < 0.001 by an unpaired two-tailed Welch’s t-test. **(F)** ChIPseeker annotation of H3K4me1 consensus peaks in young, old, geriatric FISCs. **(G)** Metaplot of H3K4me1 signal over super-enhancers (top) and typical enhancers (bottom) in young, old, and geriatric FISCs. *** p < 0.001 by an unpaired two-tailed Welch’s t-test. **(H)** Heatmap showing Spearman correlation coefficients of KR normalized Hi-C matrices, clustered by correlation distance. **(I)** Schematic of Activity-by-Contact (ABC) enhancer prediction model integrating ATAC-seq, H3K27ac CUT&RUN, and Hi-C contact information. **(J)** Genome browser snapshots showing ATAC, H3K27ac, H3K4me1 signal, and Hi-C loops at the *Sharpin* (inflammatory), *Unc13b* (synaptic), and *Nfatc1* (osteoblast differentiation) loci. **(K)** Genome browser snapshots showing ATAC, RNA-seq, H3K27ac, and H3K4me1 signal coverage across two collagen gene loci: *Col1a1* (top) and *Col3a1* (bottom). For panels (A-G and K), both males and females were considered, for (H-J) Hi-C data was from males only.
